# Long-Term Energy Consumption Minimization Based on UAV Joint Content Fetching and Trajectory Design†

**DOI:** 10.3390/s25030898

**Published:** 2025-02-02

**Authors:** Elhadj Moustapha Diallo, Rong Chai, Abuzar B. M. Adam, Gezahegn Abdissa Bayessa, Chengchao Liang, Qianbin Chen

**Affiliations:** 1 School of Communications and Information Engineering, Chongqing University of Posts and Telecommunications, Chongqing 400065, China; l202110011@stu.cqupt.edu.cn (E.M.D.); l202010006@stu.cqupt.edu.cn (G.A.B.); liangcc@cqupt.edu.cn (C.L.); cqb@cqupt.edu.cn (Q.C.); 2 Interdisciplinary Centre for Security, Reliability and Trust (SnT), University of Luxembourg, 1855 Luxembourg, Luxembourg; abuzar.babikir@uni.lu

**Keywords:** unmanned aerial vehicles (UAVs), UAV trajectory design, content fetching, content placement, power allocation

## Abstract

Caching the contents of unmanned aerial vehicles (UAVs) could significantly improve the content fetching performance of request users (RUs). In this paper, we study UAV trajectory design, content fetching, power allocation, and content placement problems in multi-UAV-aided networks, where multiple UAVs can transmit contents to the assigned RUs. To minimize the energy consumption of the system, we develop a constrained optimization problem that simultaneously designs UAV trajectory, power allocation, content fetching, and content placement. Since the original minimization problem is a mixed-integer nonlinear programming (MINLP) problem that is difficult to solve, the optimization problem was first transformed into a semi-Markov decision process (SMDP). Next, we developed a new technique to solve the joint optimization problem: option-based hierarchical deep reinforcement learning (OHDRL). We define UAV trajectory planning and power allocation as the low-level action space and content placement and content fetching as the high-level option space. Stochastic optimization can be handled using this strategy, where the agent makes high-level option selections, and the action is carried out at a low level based on the chosen option’s policy. When comparing the proposed approach to the current technique, the numerical results show that it can produce more consistent learning performance and reduced energy consumption.

## 1. Introduction

Unmanned aerial vehicles (UAVs), commonly referred to as drones, are anticipated to be a major component of future communication networks [1,2]. UAVs can act as aerial base stations (BSs) to increase network capacity and coverage, especially in areas with inadequate infrastructure or high demand. They are adaptable and can provide short-term connectivity for events, disaster recovery operations, and isolated regions [3,4].

In recent years, content provisioning services have grown rapidly due to the increasing popularity of multimedia and video applications [5]. Content caching technology is a potential solution to meet this demand. By maintaining popular content at geographically distributed servers, such as cellular system BSs, caching technology can improve data delivery efficiency [6,7,8].

Designing UAV trajectories and resource allocation strategies is crucial for UAV-enabled wireless networks. These efforts can enhance system performance, ensure effective data delivery, and maintain dependable communication links [9].

### 1.1. Related Work

This subsection provides an overview of the relevant research, including joint resource allocation and UAV trajectory planning in UAV-assisted networks and joint caching placement and UAV trajectory design.

#### 1.1.1. Joint Resource Allocation and UAV Trajectory Planning Problem in UAV-Assisted Networks

Recent work on UAV-assisted networks has focused on issues related to UAV trajectory planning and resource allocation [10,11,12,13,14,15,16,17,18,19,20,21,22].

Resource allocation and UAV trajectory planning strategies were designed by the authors of [10,11] to maximize system capacity or throughput. In [10], by using the internet of remote things (IoRT), the authors propose a technique for node scheduling, power control, and UAV trajectory planning to maximize system capacity while considering the data transmission of smart devices. In [11], the authors propose a strategy that combines UAV trajectory planning, subchannel allocation, power allocation, and communication modes to increase user throughput near the cell edge while ensuring user fairness. Although the authors of [10,11] consider throughput, they fail to address data transmission time, which is very important.

In order to reduce data transmission time, the work in [12,13,14] addresses resource allocation and UAV trajectory design. The work in [12] discusses the difficulties of deploying UAVs to collect data in urban environments. The authors propose a suboptimal method that simultaneously optimizes UAV trajectory, user scheduling, and subcarrier assignment to minimize data transmission time. However, the approach does not consider the energy consumption aspect of the UAVs. The problem of UAV trajectory and resource allocation is framed in [13] as a time consumption minimization problem, and it is solved by applying the block co-ordinate descent (BCD) algorithm. Although this method is efficient for time minimization, it does not account for varying channel conditions and energy efficiency. In order to meet the quality of service requirements of all user equipment (UE) while reducing the serving duration of a UAV, the authors of [14] address the trajectory design and resource allocation challenges for UAV-aided communications. They develop a deep reinforcement learning (DRL) algorithm based on proximal policy optimization (PPO). Although the authors develop DRL-based methods, they do not sufficiently focus on optimizing energy consumption alongside service duration.

In addition to the intended high transmission rate and shorter serving time, energy consumption is a major concern, particularly for energy-sensitive networks [15,16,17,18,19,20,21,22]. To reduce energy consumption, the authors of [15,16,17,18] designed UAV trajectory and resource allocation strategies. The authors of [15] examined the problem of joint resource allocation and UAV trajectory planning for UAV-assisted wireless networks using non-orthogonal multiple access (NOMA) for uplink communications. They propose a user association, power allocation, and UAV trajectory design strategy to minimize overall energy consumption. In [16], the authors frame the problem of UAV trajectory design, transmit power, joint task offloading ratio, and computing resource allocation as a total energy consumption minimization problem. A top-layered strategy based on DRL principles was proposed to design the UAV trajectory, with an underlying algorithm to optimize the multi-domain resource allocation problem. The authors of [17] aim to minimize the total energy consumption of UAV-enabled data-gathering systems by optimizing the trajectory, clustering, and hovering techniques of UAVs. According to [18], bit allocation, transmit power, CPU frequency, bandwidth allocation, and UAV trajectory design are taken into account while optimizing the weighted sum energy consumption of UAVs.

Although the research work in [15,16,17,18] reduces energy consumption, the reduction in energy may incur low energy efficiency. The authors of [19,20,21,22] develop resource allocation and UAV trajectory optimization methods to maximize energy efficiency (EE). The research in [19] presents an energy-efficient data-gathering technique for UAV-assisted ocean monitoring networks. The authors aim to maximize EE by jointly optimizing the transmit power of buoys and sensors, transmission scheduling, and UAV trajectory. In [20], the authors simultaneously optimize power allocation, user grouping, and UAV trajectory. A layerwise quantum-based DRL method is applied to address the problem. In [21], an efficient EE optimization problem for a cognitive UAV communication system is studied, where a moving UAV reuses the spectrum of a ground primary user to send acquired data to a leading UAV. To maximize the system’s EE, the authors propose a combined approach for resource allocation and UAV trajectory design. In [22], the authors develop a joint problem of resource allocation and UAV trajectory planning for system EE optimization. The problem is formulated as a mixed-integer nonlinear programming (MINLP) problem and involves transmit power, subchannel allocation, UAV trajectory, and speed control. It is divided into two subproblems and addressed iteratively.

#### 1.1.2. Caching Placement and UAV Trajectory Design

Recently, researchers have examined the caching placement and UAV trajectory design problem [23,24,25,26,27,28,29,30,31,32,33,34,35].

In [23,24,25,26,27,28], the authors designed UAV trajectory and caching placement to maximize throughput. The joint cache placement and UAV trajectory planning problem is addressed in [23] to increase throughput, where a two-timescale DRL algorithm is proposed to solve the problem. While the method is effective in maximizing throughput, it does not explicitly consider the associated energy consumption or the practical constraints of real-world deployments. In [24], the authors jointly optimize transmit power allocation, cache placement, and UAV trajectory design in time division multiple access (TDMA) networks to maximize throughput. However, the method fails to fully address dynamic changes in the network, which could lead to suboptimal performance in fast-evolving environments. In [25], the problem of throughput maximization is formulated for cache placement, resource allocation, and UAV trajectory planning and is solved using BCD and successive convex approximation techniques. To enhance overall network throughput, the study in [26] develops a co-operative content distribution and UAV trajectory planning strategy. For vehicular networks, the authors of [27] develop a hybrid caching and trajectory optimization method to maximize overall network throughput. In cache-enabled UAV networks, the authors of [28] study a radio resource control and trajectory design problem. They propose an actor-critic-based online reinforcement learning (RL) system to simultaneously optimize transmit power, UAV trajectory, and cache content scheduling to maximize throughput.

To maximize the system secrecy rate, the authors of [29,30,31] develop UAV trajectory and cache placement strategies. In [29], the authors discuss secure transmission in a cache-enabled UAV-relaying wireless network. In the presence of terrestrial eavesdroppers, the primary objective of this work is to optimize the minimum secrecy rate by simultaneously optimizing cache placement and UAV flight trajectory. In [30], the minimum secrecy rate is maximized over a finite time by jointly optimizing cache placement, power control, and UAV trajectory in the presence of hostile eavesdroppers. The authors of [31] optimize the number of UAVs, their 3D placements, and the cache placement probability of contents to improve the secure cache throughput of internet of things mobile devices.

In [32], the authors study energy-efficient content fetching strategies in cache-enabled device-to-device (D2D) networks. They jointly formulate a content fetching and caching problem to minimize the network’s overall energy consumption. An actor-critic RL algorithm is proposed to optimize the content fetching decisions while ensuring efficient resource utilization. The research work in [33,34] designs joint user association, cache location, UAV trajectory, and transmit power to minimize the users’ overall content acquisition latency. While effective in reducing latency, the methods do not fully consider energy consumption, which is a key factor in the sustainability of UAV networks. To maximize the cache utility of all files in the network, an iterative strategy based on matching and dynamic programming is proposed in [35]. In a D2D communication system, the authors investigate the joint optimization of UAV flight trajectory and the locations of users and UAVs.

The above studies focus on throughput maximization and cache placement but often overlook key factors such as energy consumption and dynamic system conditions. The reliance on static models and approximations, alongside the lack of real-time adaptability, underscores the need for energy-aware and dynamic UAV trajectory and caching solutions.

### 1.2. Motivation and Contributions

In recent years, a large-scale investigation of resource allocation, UAV trajectory design, and caching placement has been conducted. Yet, the existing work [10,11], as well as [23,24,25,26,27,28], mainly develops joint strategies to optimize throughput and fails to address energy consumption extensively. However, energy consumption is a significant issue, especially for UAV-enable networks that have limited energy sources. Although the authors of [19,20,21,22] study energy consumption optimization, the majority of their studies take a static approach. However, in practical scenarios, the data transmission and channel characteristics may involve dynamic change, so designing efficient resource allocation and UAV trajectory to address environmental change is important. The research work in [23,32,33,34] develops DRL-based methods to determine UAV trajectory and a resource allocation strategy in dynamic scenarios; however, the formulated Markov decision process (MDP) may consist of a large action space, which may cause complexity and low accuracy.

In this article, we consider a content fetching delivery problem in a multi-UAV-aided network, where request users (RUs) have certain content requests and UAVs are capable of offering content delivery to the RUs. In order to enhance the content fetching performance of RUs, we compute the total energy consumption of the system and formulate the joint UAV trajectory design, content fetching, transmit power allocation, and content placement problem as an energy consumption minimization. Since the formulated problem is mixed-integer nonlinear programming (MINLP), which cannot be addressed conveniently, the problem is modeled as a semi-MDP (SMDP). To address the SMDP optimization problem, we propose an option-based hierarchical DRL (OHDRL) framework.

The main contributions of this paper are summed up as follows:

- In this paper, we investigate the content delivery problem in a multi-UAV-aided network, where multiple UAVs collaborate to serve the content requests of RUs distributed across a target area. To address the energy consumption of the system, we formulate the joint UAV trajectory design, transmit power allocation, content fetching, and content placement problem as a constrained energy consumption minimization problem.
- The formulated problem is a MINLP, which is difficult to solve using conventional methods. To tackle this difficulty, we model the problem as an SMDP. In particular, to model the state of the SMDP, we take into account the co-ordinates of the UAVs; the channel state information; the remaining amount of data of the UAVs and RUs; and the caching capacity. The action space is formulated by taking into account the flying distances and directions of UAVs; the flight and hovering variable; and the transmit power of UAVs and BSs. To model the option space, we take into account the content fetching strategies of RUs and UAVs and the content placement strategy of UAVs. We introduce penalty factors to ensure optimization constraints and formulate the reward as the combination of the objective function and penalty factors.
- To enable dynamic information interaction in high dimensional states and action spaces, we propose an OHDRL-based algorithm to handle the sparse reward and non-stationary problem. In the proposed OHDRL-based framework, the original action space is divided into high-level and low-level. Specifically, we define content fetching and content placement strategies as high-level option space and define trajectory design and resource allocation strategies as low-level action space. During a specific time period, the agents make higher-level option selections, and the actions are carried out at a lower level in accordance with the option’s internal policy. The joint strategy can be obtained by combining off-policy within options and on-policy between options.
- To evaluate the performance of the proposed algorithm, we use Python to build neural networks. In the OHRDL-based algorithm, we choose the network parameters reasonably so that the algorithm convergence can be achieved and content fetching and UAV trajectory strategies can be obtained. The simulation results demonstrate that the proposed OHDRL-based algorithm improves the learning efficiency of complex tasks. In terms of energy consumption, our proposed OHDRL-based approach performs better than the reference algorithms.

The rest of the paper is structured as follows. The system model, which comprises the network model and channel model, is introduced in Section 2. In Section 3, the energy consumption optimization problem is formulated. We develop an algorithm based on the OHDRL framework in Section 4 to solve the formulated problem. Section 5 is dedicated to the performance evaluation of the proposed strategy. Section 6 concludes the paper. The parameters and symbols of the main notations appearing in this paper are shown in Table 1.

## 2. System Model

In this section, we discuss the network model and channel model.

### 2.1. Network Model

**Figure 1:**
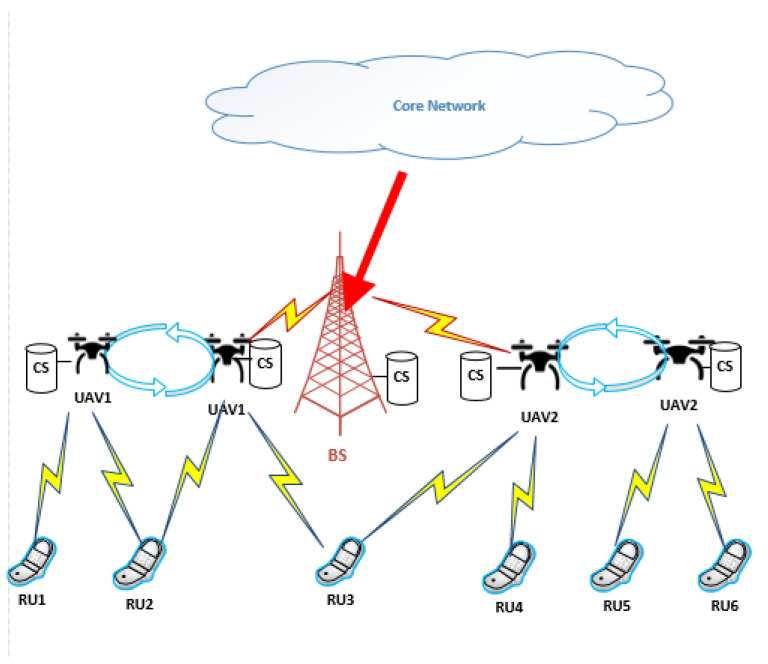
System model.

As shown in Figure 1, we consider content delivery in a UAV-assisted network composed of a BS and a number of UAVs and RUs. Suppose the RUs have certain content requests, and the BS retrieves the requested content from the core network through wired backhaul links and pre-caches the contents on its collocated content server. Considering the scenario where the RUs might be far from the BS, resulting in undesired transmission performance, we employ UAVs as mobile BSs that provide content delivery service to the RUs.

To enhance content delivery performance, we assume that UAVs fetch certain contents from the BS, store them in their local caches, and transmit them to the RUs upon request. Assuming that the UAVs are allowed to fetch and send contents simultaneously, self-interference may exist. For simplicity, we assume that by using particular schemes, the self-interference can be canceled. For data delivery, UAVs are assumed to hover at fixed locations during data transmission to ensure stable communication with the RUs. In the considered system, we apply an orthogonal frequency division multiple access (OFDMA) scheme where multiple RUs may access the UAVs using orthogonal subcarriers. We suppose that the entire bandwidth is divided into a number of equal-length bandwidth subchannels. Let B denote the bandwidth of each subchannel and ω denote the number of subchannels. It is assumed that the UAVs fly from their initial positions to certain target areas in order to serve the RUs and then return to their starting places after the content delivery service is finished. Let I and M represent the number of RUs and the number of UAVs, respectively; we denote RU*_i_* as the i-th RU and U*_m_* as the m-th UAV, 1≤i≤I, 1≤m≤M.

We divide the system time T into equal-length time slots for simplicity. The length of each time slot is denoted by τ, and the total number of time slots is represented by N, i.e., T=Nτ. It is assumed that the positions of the UAVs remain fixed during each time slot. The location of U*_m_* at time slot t is given as $q_{m}\left(t\right)\;=\;\left(x_{m}\left(t\right),\;\;y_{m}\left(t\right),\;H\right)$, where H is the fixed flight altitude of U*_m_*. Let ${\tilde{q}}_{i}\;$$=\;\left({\tilde{x}}_{i},\;\;{\tilde{y}}_{i},\;0\right)$ and $q_{b}=\;\left(x_{b},\;y_{b},\;0\right)$ represent the co-ordinates of RU*_i_* and the BS, respectively.

Assume that the RUs can obtain content from a limited content library without loss of generality. Let F*_k_* represent the k-th content, let $L_{k}$ denote the size of F*_k_*, 1≤k≤K, and let K denote the total number of contents. A binary variable $\mu_{i,k}\in 0,1$ is introduced to indicate whether RU*_i_* requests content, F*_k_*. More specifically, each RU demands content based on its popularity and individual preferences. Thus, $\mu_{i,k}$ is treated as a constant and is predetermined in this work. For simplicity, we assume that each RU requests only a single piece of content, i.e., $\sum_{k=1}^{K}\mu_{i,k}=1$, ∀i. Let $\delta_{m,k}$ represent the content placement variable of U*_m_*, $\delta_{m,k}=1$ if F*_k_* is cached in U*_m_*, and $\delta_{m,k}=0$; otherwise, ∀m,k.

### 2.2. Channel Model

In this subsection, we discuss the channel models of BS-UAV links and UAV-RU links and then formulate the data rates of the transmission links.

#### 2.2.1. Channel Model of BS-UAV Links

Since the line-of-sight (LoS) link dominates the UAV-to-ground channel, UAV communications typically have better channel conditions compared to terrestrial communications. Let $h_{m}\left(t\right)$ be the channel gain between the BS and U*_m_* at time slot t, which can be written as

(1) $$h_{m}\left(t\right)=\beta {\left(d_{m}\left(t\right)\right)}^{-\alpha }10^{\eta_{e}}/10,$$

where β denotes the reference channel gain at a distance of 1 m, α denotes the path loss coefficient, $\eta_{e}∼\;N\left(0,\;\sigma_{e}^{2}\right)$ is modeled as a Gaussian random variable, $e\;\in \;\left\{\mathrm{LoS},\;\mathrm{NLoS}\right\}$ is the propagation parameter, and $d_{m}\left(t\right)$ represents the distance between BS and U*_m_* at time slot t, which can be formulated as

(2) $$d_{m}\left(t\right)\;=\;\sqrt{{\left(x_{m}\left(t\right)-x_{b}\right)}^{2}\;+{\left(y_{m}\left(t\right)-y_{b}\right)}^{2}\;+\;H^{2}\;}.$$

Let $R_{m}\left(t\right)$ represent the data transmission rate of the link between BS and U*_m_* at time slot t, which can be written as

(3) $$R_{m}\left(t\right)=\;B\log_{2}\left(1+\frac{P_{m}^{t}\left(t\right)h_{m}\left(t\right)}{\sigma^{2}}\right),$$

where $P_{m}^{t}\left(t\right)$ denotes the transmit power of the BS when sending contents to U*_m_* at time slot t, and $\sigma^{2}$ denotes the noise power.

#### 2.2.2. Channel Model of UAV-RU Links

Let $h_{m,i}\left(t\right)$ represent the channel gain between U*_m_* and RU*_i_* at time slot t, which can be formulated as

(4) $$h_{m,i}\left(t\right)=\beta {\left(d_{m,i}\left(t\right)\right)}^{-\alpha }10^{\eta_{e}}/10,$$

where $d_{m,i}\left(t\right)$ is the distance between U*_m_* and RU*_i_* at time slot t, which can be written as

(5) $$d_{m,i}\left(t\right)=\;\sqrt{{\left(x_{m}\left(t\right)-x_{i}\right)}^{2}\;+{\left(y_{m}\left(t\right)-y_{i}\right)}^{2}\;+H^{2}\;}.$$

Let $R_{m,i}\left(t\right)$ denote the data transmission rate of the link between U*_m_* and RU*_i_* at time slot t, which can be formulated as

(6) $$R_{m,i}\left(t\right)=\;B\log_{2}\left(1+\frac{P_{m,i}^{t}\left(t\right)h_{m,i}\left(t\right)}{\sigma^{2}}\right),$$

where $P_{m,i}^{t}\left(t\right)$ represents the transmit power of U*_m_* while delivering the content to RU*_i_* at time slot t.

## 3. Energy Consumption Optimization Problem

In this section, we examine the energy consumption of RUs in retrieving content from UAVs and formulate the problem of UAV trajectory, content fetching, and power allocation as an energy consumption minimization problem.

### 3.1. Objective Function

The deployment of UAVs benefits the RUs by improving content fetching performance. However, UAVs are energy-sensitive devices. In this subsection, we formulate the energy consumption of the system, which is defined as the total energy consumed during the flying, hovering, and data transmission of the UAVs. Consequently, the total energy consumption of the system at time slot t, denoted by E(t), can be formulated as follows:

(7) $$E\left(t\right)=E^{f}\left(t\right)+E^{h}\left(t\right)+E^{t}\left(t\right),$$

where $E^{f}\left(t\right)$ and $E^{h}\left(t\right)$ are, respectively, the flight and hovering energy consumption of the UAVs at time slot t, and $E^{t}\left(t\right)$ represents the energy consumption of the UAVs resulting from data transmission at time slot t. $E^{f}\left(t\right)$ can be computed as

(8) $$E^{f}\left(t\right)=\sum_{m=1}^{M}\theta_{m}\left(t\right)\;E_{m}^{f}\left(t\right),$$

where $\theta_{m}\left(t\right)$ denotes the flight variable of U*_m_*; if U*_m_* flies at time slot t, $\theta_{m}\left(t\right)=1$; if U*_m_* hovers at a certain position at time slot t, $\theta_{m}\left(t\right)=0$. $E_{m}^{f}\left(t\right)$ denotes the flight energy consumption of U*_m_* at time slot t, which is modeled as

(9) $$E_{m}^{f}\left(t\right)=P_{m}^{f}\left(t\right)\tau ,$$

where $P_{m}^{f}\left(t\right)$ indicates the propulsion power of U*_m_* during flight at time slot t, which can be formulated as

(10) $$P_{m}^{f}\left(t\right)=P^{0}\left(1+\frac{3v_{m}^{2}\left(t\right)}{v_{\mathrm{tip}}^{2}}\right)+\frac{1}{2}f_{0}\rho sAv_{m}^{3}\left(t\right)+P^{1}\;{\left(\sqrt{1\;+\frac{v_{m}^{4}\left(t\right)}{4v_{0}^{4}}}-\frac{v_{m}^{2}\left(t\right)}{2v_{0}^{2}}\right)}^{1/2},$$

where $P^{0}$ and $P^{1}$ are constants representing, respectively, the blade profile power and induced power in the hovering status; $v_{m}\left(t\right)$ denotes the velocity of U*_m_* at time slot t, $v_{\mathrm{tip}}$ represents the tip speed of the rotor blade, and $v_{0}$ is the mean rotor induced velocity in the hovering status; $f_{0}$ and s are the fuselage drag ratio and rotor solidity, respectively; ρ and A denote the air density and rotor disc area.

$E^{h}\left(t\right)$ in (7) can be written as follows

(11) $$E^{h}\left(t\right)=\sum_{m=1}^{M}\left(1-\theta_{m}\left(t\right)\right)\left(P^{0}+P^{1}\right)\tau ,$$

$E^{t}\left(t\right)$ in (7) can be formulated as

(12) $$E^{t}\left(t\right)=\sum_{m=1}^{M}E_{m}^{t}\left(t\right),$$

where $E_{m}^{t}\left(t\right)$ is the transmission energy consumption of U*_m_* at time slot t, which can be calculated as

(13) $$E_{m}^{t}\left(t\right)=\sum_{i=1}^{I}\alpha_{m,i}\left(t\right)E_{m,i}^{t}\left(t\right)+\phi_{m,k}\left(t\right)E_{b,m}^{t}\left(t\right)$$

where $\alpha_{m,i}\left(t\right)$ indicates the content fetching variable of RU*_i_*. If RU*_i_* is receiving contents from U*_m_* at time slot t, then $\alpha_{m,i}\left(t\right)=1$; otherwise, $\alpha_{m,i}\left(t\right)=0$, and $\phi_{m,k}\left(t\right)$ indicates the content fetching variable of U*_m_*. If U*_m_* fetches content k from the BS at time slot t, $\phi_{m,k}\left(t\right)=1$; otherwise, $\phi_{m,k}\left(t\right)=0$. $E_{m,i}^{t}\left(t\right)$ indicates the transmission energy consumption of U*_m_* while delivering the contents to RU*_i_* at time slot t, which is computed as

(14) $$E_{m,i}^{t}\left(t\right)=\;P_{m,i}^{t}\left(t\right)\;T_{m,i}\left(t\right),$$

where $T_{m,i}\left(t\right)$ indicates the transmission delay when U*_m_* sends contents to RU*_i_* at time slot t. Let $S_{i}\left(t\right)$ indicate the remaining amount of data that RU*_i_* fetches from UAVs at time slot t, which can be written as

(15) $$S_{i}\left(t\right)=\max\left\{\sum_{k=1}^{K}\mu_{i,k}L_{k}-\sum_{m=1}^{M}\sum_{t_{0}=1}^{t-1}\alpha_{m,i}\left(t_{0}\right)R_{m,i}\left(t_{0}\right)T_{m,i}\left(t_{0}\right),0\right\}$$

$T_{m,i}\left(t\right)$ can further be formulated as

(16) $$T_{m,i}\left(t\right)=\left\{\begin{array}{cc} \tau ,\; & \mathrm{if}\;\;\frac{S_{i}\left(t\right)}{R_{m,i}\left(t\right)}\geq \tau \\ \frac{S_{i}\left(t\right)}{R_{m,i}\left(t\right)},\; & \mathrm{otherwise}. \end{array}\right.$$

In (13), $E_{b,m}^{t}\left(t\right)$ indicates the transmission energy consumption of the BS while delivering the uncached contents to U*_m_* at time slot t, which is formulated as

(17) $$E_{m}^{t}\left(t\right)=\;P_{m}^{t}\left(t\right)\;T_{m}\left(t\right),$$

where $T_{m}\left(t\right)$ indicates the transmission delay during data transfer from the BS to U*_m_*. Let ${\bar{S}}_{m}\left(t\right)$ indicate the remaining amount of data that U*_m_* fetches from BS at time slot t, which can be expressed as

(18) $${\bar{S}}_{m}\left(t\right)=\max\left\{\sum_{k=1}^{K}\left(1-\delta_{m,k}\right)L_{k}-\sum_{m=1}^{M}\sum_{t_{0}=1}^{t-1}\phi_{m,k}\left(t_{0}\right)R_{m}\left(t_{0}\right)T_{m}\left(t_{0}\right),0\right\}$$

where $\phi_{m,k}$ is the content fetching variable of U*_m_*, $T_{m}\left(t\right)$, which can be calculated as

(19) $$T_{m}\left(t\right)=\left\{\begin{array}{cc} \tau ,\;\; & \;\;\;\;\mathrm{if}\;\;\;\;\frac{{\bar{S}}_{m}\left(t\right)}{R_{m}\left(t\right)}\geq \tau \\ \frac{{\bar{S}}_{m}\left(t\right)}{R_{m}\left(t\right)},\;\; & \mathrm{otherwise}. \end{array}\right.$$

### 3.2. Optimization Constraints

In this subsection, we outline the optimization constraints that must be satisfied to design a joint UAV trajectory and content fetching strategy.

#### 3.2.1. Content Fetching Constraints

We assume that at any given time slot, only one RU can be served by a single UAV; thus, we obtain

(20) $$C1:\sum_{m=1}^{M}\alpha_{m,i}\left(t\right)\leq 1,\forall i,t.$$

To enable a multi-subchannel content fetching scheme, we assume that one UAV may serve different RUs with different subchannels, i.e.,

(21) $$C2:\sum_{i=1}^{I}\alpha_{m,i}\left(t\right)\leq \omega ,\forall m,t.$$

We assume that at a given time slot, only one UAV can fetch the contents from the BS; we obtain

(22) $$C3:\sum_{m=1}^{M}\phi_{m,k}\left(t\right)\leq 1,\forall t.$$

#### 3.2.2. Transmission Rate Constraint

In order to ensure the effective transfer of contents, we assume that the data transmission rate should be greater than a threshold, i.e.,

(23) $$C4:{\bar{R}}_{i}\left(t\right)\geq \sum_{m=1}^{M}\sum_{k=1}^{K}\alpha_{m,i}\left(t\right)\mu_{i,k}R_{k}^{\mathrm{th}},$$

where $R_{k}^{\mathrm{th}}$ denotes the data transmission rate threshold of F*_k_*, and ${\bar{R}}_{i}\left(t\right)$ represents the data rate of RU*_i_*, which can be written as

(24) $${\bar{R}}_{i}\left(t\right)=\sum_{m=1}^{M}\alpha_{m,i}\left(t\right)R_{m,i}\left(t\right)$$

#### 3.2.3. Transmit Power Constraints

The transmit power is limited by the maximum transmit power, i.e.,

(25) $$C5:\sum_{i=1}^{I}\alpha_{m,i}\left(t\right)P_{m,i}^{t}\left(t\right)\leq P_{m}^{\max},\forall m,t,$$

where $P_{m}^{\max}$ represents the maximum transmit power of U*_m_*.

(26) $$C6:\sum_{m=1}^{M}\phi_{m,k}\left(t\right)P_{m}^{t}\left(t\right)\leq P^{\max},\forall t,$$

where $P^{\max}$ denotes the maximum transmit power of the BS.

#### 3.2.4. Flight Trajectory Constraints

The flight distance of UAVs in adjacent time slots is limited by the following constraint, i.e.,

(27) $$\begin{array}{c} C7:{∥q_{m}\left(t+1\right)-q_{m}\left(t\right)∥}_{2}\leq v_{m}^{\max}\tau ,\forall m,t \end{array}$$

where $v_{m}^{\max}$ is the maximum speed of U*_m_*.

In order to prevent collisions between multiple UAVs, we have the following constraint:

(28) $$C8:{∥q_{m}\left(t\right)-q_{m^{\prime }}\left(t\right)∥}_{2}\geq l_{\mathrm{th}},\forall m\neq m^{\prime },t,$$

where $l_{\mathrm{th}}$ is the safe distance of UAVs.

We assume each UAV has the same flight cycle with the initial and end locations as

(29) $$C9:q_{m}\left(0\right)=q_{m}\left(T\right),\forall m.$$

#### 3.2.5. Content Placement Constraint

Due to the restricted cache space, the contents cached in UAVs should adhere to the maximum cache capacity constraint, i.e.,

(30) $$C10:\;\sum_{k=1}^{K}\delta_{m,k}L_{k}\leq \;\psi_{m},\forall m,$$

where $\psi_{m}$ is the maximum cache capacity of U*_m_*.

### 3.3. Optimization Problem Formulation

Given the constraints of the content fetching, transmit power, and flight trajectory of the UAVs, the UAV trajectory design and content fetching problem is formulated as an energy consumption minimization problem, which can be written as

(31) $$\begin{array}{c} \min_{q_{m}\left(t\right),P_{m,i}^{t}\left(t\right),P_{m}^{t}\left(t\right),\alpha_{m,i}\left(t\right),\phi_{m,k}\left(t\right),\delta_{m,k}}\frac{1}{T}\sum_{t=1}^{T}\;E(t)\\ \;\;\;\;\;\;\;\;\;\;\;\;\;\;\;\;\;\;\;\;\;\;\;\;\;\;\;\;\;s.t.\;\;C1-C10. \end{array}$$

## 4. Solution to the Optimization Problem

Since the optimization problem described in (31) is MINLP, it is difficult to solve optimally using traditional approaches. In order to address this challenge, we first formulated the problem as an SMDP and, subsequently, proposed an OHDRL framework to determine UAV trajectory, content fetching, and a power allocation strategy.

### 4.1. An Introduction to SMDP

MDP is a probabilistic decision-making instrument based on the Markov property theory. It is used to determine optimal decisions for dynamic systems while taking into account both their operating environment and current state. MDPs operate on a rigid framework of fixed, discrete time steps, where state transitions occur immediately after each time step. At each time step, an agent, also known as a decision-maker, examines the current state of the system, chooses an action from a set of available actions, and moves to a new state according to the chosen action’s stochastic transition probabilities. As a result of its actions, the agent is rewarded numerically, and the objective is to identify an optimal policy that prescribes the best action to take in each state in order to maximize the cumulative expected rewards over time.

In an MDP, there are several fundamental components and concepts. Namely, states represent the different situations or configurations of the environment, and the set of all possible states is denoted as S. Actions represent the choices or decisions that the agent can make within the environment, and the set of all possible actions is denoted as A. The reward function R assigns a numerical value to each state-action pair, indicating the immediate desirability of taking a specific action in a given state. The reward function serves as a key component in MDPs, enabling the agent to learn and modify its policies to successfully accomplish its long-term objectives. Transition probabilities describe a function that defines the probability of moving from one state to another after taking a specific action. They describe how the environment responds to the agent’s actions and incorporate uncertainty.

**Figure 2:**
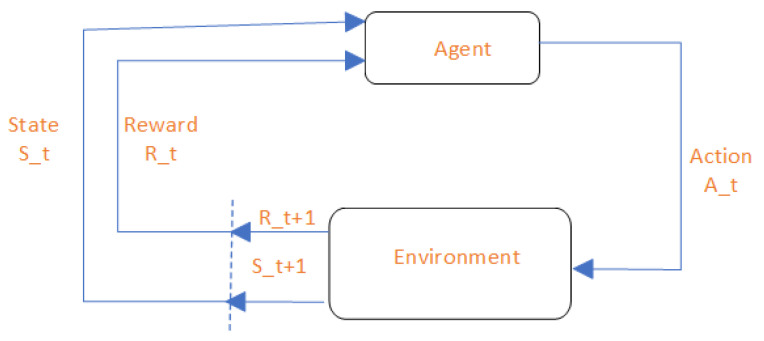
A graphical representation of the MDP model.

The policy function π guides the agent’s behavior and determines its actions in response to the environment’s stochastic transitions. In an MDP, there are different types of policies, including deterministic and stochastic policies. Deterministic policies select a single action for each state, while stochastic policies assign probabilities to each action, allowing for exploration and handling uncertainty. The value function V(s) in an MDP is a fundamental concept that quantifies the expected return an agent can achieve starting from a given state. It is important in decision-making because it represents the long-term desirability of a state. Specifically, the value function provides a way to rank states based on their expected cumulative rewards, guiding the agent towards more favorable states and actions. See Figure 2.

As an extension of MDPs, an SMDP is an advanced mathematical framework that combines the concepts of both MDPs and semi-Markov processes. SMDPs are designed to model decision-making problems where actions are taken by an agent in an environment with stochastic state transitions. In contrast to traditional MDPs, the time spent in each state is modeled by a more general distribution [36]. SMDPs introduce flexibility by allowing variable time intervals between state transitions, accommodating scenarios where actions may require different amounts of time to complete. Additionally, in SMDPs, states can endure for variable durations, which are stochastic and follow specific probability distributions, offering a more nuanced representation of how states evolve over time.

Similar to traditional MDPs, an SMDP can also be characterized by states, actions, rewards, and probabilities. Unlike an MDP, in an SMDP, the state transition probabilities depend not only on the current state and the chosen action but also on the time spent in the current state. This accounts for the variable duration of time spent in a state before transitioning to a new one. Rewards can be time-dependent, and an agent may accumulate rewards over time within a state.

SMDPs are preferred over standard MDPs in specific scenarios. They excel when actions within an environment take varying amounts of time to complete, as they can accurately model this temporal variability, resulting in a more realistic problem representation. Additionally, SMDPs are well-suited for problems with continuous or semi-continuous state spaces, as they can effectively handle both variable durations and continuous state changes [37]. When decision-making involves complex sequences of actions with varying time constraints, SMDPs provide a more precise depiction of the decision-making process [38].

### 4.2. An Introduction to DRL and OHDRL

In this subsection, we present a brief overview of DRL and OHDRL.

#### 4.2.1. An Overview of DRL

DRL is a subdiscipline of artificial intelligence and machine learning that focuses on teaching machines to learn and make decisions in complex environments. By combining RL with deep learning techniques, DRL can tackle challenging problems that involve sequential decision-making in MDPs. The DRL framework operates at the primitive action level and does not explicitly incorporate temporal abstractions or higher-level actions. DRL typically uses a flat, single-level structure for decision-making, where actions are selected at the atomic level based on the current state. In DRL, the agent must learn to manage the complexity of tasks at the atomic action level, which can be challenging for tasks with long time horizons or large action spaces. Learning can be less sample-efficient in DRL, particularly for tasks with high-dimensional state spaces and complex dynamics.

In order to evaluate the Q-values, DQN, which combines deep learning techniques with Q-learning, is proposed. In the DQN framework, two important networks are crucial. The prediction network is responsible for predicting the Q-values for different actions in a given state. These Q-values indicate the expected cumulative future rewards for taking a specific action in the current state. On the other hand, the target network serves as a stable reference for generating target Q-values during the training process. It provides a more consistent and less volatile set of Q-value targets compared to the Q-network. Let Q(s,a;θ) and $Q\;(s^{\prime },a^{\prime };\;\bar{\theta })$ represent the Q-values of the prediction network and the target network, respectively. Q(s,a;θ) can be computed as

(32) $$\begin{array}{c} Q\;\left(s,a;\;\theta \right)\;=\;r\left(s,a\right)+\gamma \sum_{s^{\prime }\in S}T\left(s,a,s^{\prime }\right)\left[\max_{a^{\prime }\in A}Q\;\left(s^{\prime },a^{\prime };\;\bar{\theta }\right)\;\right],\\ =E_{s^{\prime }∼T\left(s,a,s^{\prime }\right)}\left[R\left(s,a,s^{\prime }\right)+\gamma \max_{a^{\prime }\in A}Q\;\left(s^{\prime },a^{\prime };\;\bar{\theta }\right)\right], \end{array}$$

where θ represents the weights of the prediction network, which are updated after each iteration; $\bar{\theta }$ represents the weights of the target network, which are periodically synchronized with the parameters of the prediction Q-network; $R(s,a,s^{\prime })$ denotes the immediate reward from state s to state $s^{\prime }$ when taking action a; $T(s,a,s^{\prime })$ is the transition probability from state s to state $s^{\prime }$ when taking action a; and γ denotes the discount factor.

The mean squared error (MSE) is utilized as the loss function to optimize the prediction network parameters. The loss function calculates the difference between the predicted values (e.g., state-action values or policy probabilities) and the target values (e.g., target state-action values or advantages). It quantifies how far off the agent’s current estimates are from the desired values. This error estimation is crucial for guiding the learning process. The loss function L(θ) can be written as

(33) $$L\left(\theta \right)=E_{s^{\prime }∼T\left(s,a,s^{\prime }\right)}\left[{\left(Q\left(s,a;\theta \right)-R\left(s,a,s^{\prime }\right)-\gamma \max_{a^{\prime }\in A}Q\left(s^{\prime },a^{\prime };\bar{\theta }\right)\right)}^{2}\right].$$

#### 4.2.2. An Overview of OHDRL

OHDRL extends traditional hierarchical RL by integrating DRL techniques to address the scalability challenges of conventional DRL methods in complex tasks. This approach enhances RL practicality for long-term planning, complex environments, and high-dimensional state spaces. By structuring agents to learn and execute subpolicies, OHDRL enables tackling challenging problems that traditional RL methods struggle to solve.

In OHDRL, options are introduced as subpolicies or temporally extended actions learned and utilized at a higher level of abstraction [39]. An option is defined as a tuple $\left(\xi ,\pi_{o},\lambda_{o}\left(s\right)\right)$, where ξ is the initiation set, $\pi_{o}$ is the option’s policy, and $\lambda_{o}\left(s\right)$ represents the termination condition. The termination condition indicates the probability of an option, o, ending in state s. By explicitly modeling temporal abstractions, OHDRL simplifies complex tasks by decomposing them into subtasks represented by options. This hierarchical structuring leads to more efficient exploration and faster learning, particularly for tasks with inherent hierarchical structures [40,41].

The OHDRL architecture operates on two levels: a high-level policy and a low-level policy. The high-level policy selects options based on the current state and internal goals, which may be predefined by the task or learned during training. Once an option is chosen, the associated low-level policy determines the sequence of actions to execute until the subgoal is achieved or the termination condition is met. This dual-layer approach enables the agent to manage both long-term and short-term decision-making effectively.

In the OHDRL algorithm, the intra-option policy $\pi_{o}\left(a|s\right)$ specifies the probability of taking action, a, in state s under the current option o. $\pi_{o}\left(a|s\right)$ can be calculated as

(34) $$\pi_{o}\left(a|s\right)=\mathrm{softmax}\left(Q_{o}\left(s,a\right)\right),$$

where $Q_{o}\left(s,a\right)$ represents the option Q-value function. This function estimates the expected cumulative reward when starting in state s, executing option o, taking action a, and following the option policy $\pi_{o}$. $Q_{o}\left(s,a\right)$ can be given by

(35) $$Q_{o}\left(s,a\right)=r\left(s,a\right)+\gamma \sum_{s^{\prime }}T\left(s,a,s^{\prime }\right)\max_{a^{\prime }}Q_{o}\left(s^{\prime },a^{\prime }\right),$$

The termination function $\lambda_{o}\left(s\right)$ can be calculated as

(36) $$\lambda_{o}\left(s\right)=\mathrm{sigmoid}\left(f_{\lambda_{o}}\left(s\right)\right),$$

where $f_{\lambda_{o}}\left(s\right)$ is a learned function mapping states to values in [0,1]. The intra-option value function $V_{o}\left(s\right)$ represents the expected cumulative reward of executing option o, starting from state s and following the option’s policy until termination, which can be expressed as

(37) $$V_{o}\left(s\right)=\sum_{a}\pi_{o}\left(a|s\right)Q_{o}\left(s,a\right).$$

### 4.3. SMDP Modeling

In this subsection, we first transform the problem formulation in (31) into an SMDP and then provide an OHDRL-based algorithm to solve the optimization problem. The summary of the dimensions of the state, action, and option variables are shown in Table 2. The details of the state, action, option, and reward in the proposed OHDRL architecture are described below.

(a) State space S: The state space consists of four main components, which can be written as follows:

(38) $$S(t)\overset{\Delta }{\;=}\left\{S_{1}\left(t\right),S_{2}\left(t\right),S_{3}\left(t\right),S_{4}\left(t\right)\right\},$$

where $S_{1}\left(t\right)\overset{\Delta }{\;=}\left\{q_{m}\left(t\right)\right\}$ denotes the co-ordinates of the UAVs, $S_{2}\left(t\right)\overset{\Delta }{\;=}\left\{h_{m,i}\left(t\right),h_{m}\left(t\right)\right\}$ indicates the channel state information, $S_{3}\left(t\right)\overset{\Delta }{\;=}\left\{S_{i}\left(t\right),{\bar{S}}_{m}\left(t\right)\right\}$ represents the remaining amount of data of UAVs and RUs, and $S_{4}\left(t\right)\overset{\Delta }{\;=}\left\{\psi_{m}\right\}$ represents the caching capacity state.

(b) Option space O: The options are chosen as the content fetching strategies of RU*_i_* and U*_m_* and the content placement strategy of U*_m_*, 1≤m≤M. Accordingly, the option space can be expressed as

(39) $$O\overset{\Delta }{\;=}\left\{\alpha_{m,i}\left(t\right),\phi_{m,k}\left(t\right),\delta_{m,k}\right\}.$$

Note that choosing $\alpha_{m,i}\left(t\right)$ in the option space allows the UAV to optimize its delivery strategy and determine which RUs to serve and when to serve the RUs; choosing $\phi_{m,k}\left(t\right)$ in the option space allows the UAVs to optimize their fetching strategy and determine when the UAVs should fetch content from the BS; choosing $\delta_{m,k}$ in the option space allows for determining which content the UAVs should cache. In (39), each option has a terminal condition. Let $\lambda =\left\{\lambda_{\alpha_{m,i}\left(t\right)},\lambda_{\phi_{m}\left(t\right)},\lambda_{\delta_{m,k}}\right\}$ indicate the termination condition, where $\lambda_{\alpha_{m,i}\left(t\right)}$, $\lambda_{\phi_{m}\left(t\right)}$ and $\lambda_{\delta_{m,k}}$ are the terminal conditions of $\alpha_{m,i}\left(t\right)$, $\phi_{m}\left(t\right)$ and $\delta_{m,k}$, respectively.

(c) Action space A: The action space of the SMDP is hybrid in nature, comprising both discrete and continuous variables; A can be expressed as

(40) $$A(t)\overset{\Delta }{\;=}\left\{a_{1}\left(t\right),a_{2}\left(t\right),a_{3}\left(t\right)\right\},$$

where $a_{1}\left(t\right)$ is a discrete action representing the movement of $U_{m}$ at time slot t. It is expressed as $a_{1}\left(t\right)=\left\{\kappa_{m}\left(t\right),\varepsilon_{m}\left(t\right)\right\}$, where $\kappa_{m}\left(t\right)$ and $\varepsilon_{m}\left(t\right)$ indicate the flying distance and direction of $U_{m}$ at time slot t. $a_{2}\left(t\right)$ is a discrete action representing the operational state of the UAV, defined as $a_{2}\left(t\right)=\left\{\theta_{m}\left(t\right)\right\}$, where $\theta_{m}\left(t\right)$ determines whether $U_{m}$ is in a flying or hovering state. $a_{3}\left(t\right)$ is a continuous action representing the transmit power of the BS and $U_{m}$, defined as $a_{3}\left(t\right)=\left\{P_{m,i}^{t}\left(t\right),P_{m}^{t}\left(t\right)\right\}$. By selecting the flight action $a_{m}\left(t\right)$, the position of $U_{m}$ at slot t can be updated as

(41) $$x_{m}\left(t+1\right)=x_{m}\left(t\right)+\kappa_{m}\left(t\right)\cos\left(\varepsilon_{m}\left(t\right)\right),$$

(42) $$y_{m}\left(t+1\right)=y_{m}\left(t\right)+\kappa_{m}\left(t\right)\sin\left(\varepsilon_{m}\left(t\right)\right).$$

Note that the actions $\kappa_{m}\left(t\right)$ and $\varepsilon_{m}\left(t\right)$ should be chosen to ensure that constraints C7 and C8 hold. Given a specific option over a relatively long time scale, the actions can be chosen in a relatively short time scale. For instance, for a given content fetching strategy, the UAV trajectory can be designed for each time slot.

(d) Reward: When all the options are terminated, the agent can obtain a reward. Let R(t) represent the cumulative rewards at time slot t, which can expressed as

(43) $$R\left(t\right)=-E\left(t\right)-\varepsilon^{\mathrm{cf}}-\varepsilon^{p}-\varepsilon^{q}-\varepsilon^{\mathrm{cp}},$$

where $\varepsilon^{\mathrm{cf}}$ represents the content fetching penalty associated with constraints C1–C4, $\varepsilon^{p}$ represents the power allocation penalty associated with constraints C5 and C6, $\varepsilon^{q}$ represents the flight trajectory penalty associated with constraints C7 and C8, and $\varepsilon^{\mathrm{cp}}$ represents the content placement penalty associated with constraint C10.

In the realm of solving sequential decision-making problems, the application of OHDRL proves to be a promising framework. The optimization objective in SMDPs is typically to find a policy that maximizes the expected cumulative reward over time.

### 4.4. OHDRL Framework

Based on the modeled SMDP, we propose an OHDRL framework to determine the actions leading to long-term reward maximization. In the proposed OHDRL framework, we employ a multi-agent RL framework, where multiple agents collaborate to optimize the global system objectives. Each UAV is equipped with its own dedicated agent; however, these agents are not independent. Instead, they operate in a co-ordinated and interdependent manner to achieve a unified objective. The agents exchange critical state information, allowing them to make informed decisions that align with the overarching goals of minimizing energy consumption and enhancing operational efficiency. At time slot t, the agent selects the option $o\left(t\right)\in O$ based on state $s\left(t\right)\in S$ and the option policy π. Then, the action $a\left(t\right)\in A$ is executed. When option $o\left(t\right)$ is terminated and action $a\left(t\right)$ is executed, the reward R is received. At time slot t+1, the agent selects option $o\left(t+1\right)$ based on intra-option policy Φ and state $s\left(t+1\right)$. This procedure is repeated until the end of the process. By using the Bellman function, the option value function of the agent corresponding to the action value function on state $s\left(t\right)$ can be written as

(44) $$\begin{array}{cc} \; & Q_{\pi }\left(s\left(t\right),o\left(t\right)\right)=r^{o}\left(t\right)\\ \; & +\sum_{t+1}p^{o}\left(t,t+1\right)\sum_{o(t+1)}\pi \left(s\left(t+\left|o(t)\right|\right),o\left(t+1\right)\right)Q_{\pi }\left(s\left(t+\left|o(t)\right|\right),o\left(t+1\right)\right), \end{array}$$

where $\left|o\left(t\right)\right|$ represents the option’s time length, and $r^{o}\left(t\right)$ is the reward associated with the chosen option based on the intra-option policy Φ, which is described as follows

(45) $$\begin{array}{cc} \; & r^{o}\left(t\right)=\\ \; & \sum_{a(t)\in A}\Phi \left(s\left(t\right),a\left(t\right)\right)\left(r^{a}\left(t\right)+\sum_{s(t+1)}p^{a}\left(t,t+1\right)\left(1-\lambda \left(s\left(t+1\right)\right)r^{o}\left(t+1\right)\right)\right). \end{array}$$

In (44), $p^{o}\left(t,t+1\right)$ denotes the option transition probability, which can be expressed as

(46) $$\begin{array}{cc} \; & p^{o}\left(t,t+1\right)=\\ \; & \sum_{a(t)\in A}\Phi \left(s\left(t\right),a\left(t\right)\right)\sum_{s(t+1)}\left(p^{a}\left(t,t+1\right)\left((1-\lambda \left(s\left(t+1\right)\right)p^{o}\left(t+1\right)+\lambda \left(s\left(t+1\right)\right)\right)\right), \end{array}$$

where $\lambda \left(s\left(t+1\right)\right)$ indicates the termination condition. By using the termination condition, we define the following parameter to adjust option exploration:

(47) $$a\left(t+1\right)=a\left(t\right)-b\left(r\left(t\right)+\gamma V_{\pi }\left(s\left(t+1\right)\right)-V_{\pi }\left(s\left(t\right)\right)\right)\partial \nabla \lambda \left(s\left(t\right)\right),$$

where b indicates the positive step size; the value function can be defined as follows:

(48) $$V_{\pi }\left(s\left(t\right)\right)=\sum_{o\left(t\right)\in O}\Phi \left(s\left(t\right)|a\left(t\right)\right)Q_{\pi }\left(s\left(t\right),o\left(t\right)\right).$$

Our final goal is to find the optimal option value function $Q_{\pi }^{*}\left(s\left(t\right),o\left(t\right)\right)$, which is formulated as follows:

(49) $$Q_{\pi }^{*}\left(s\left(t\right),o\left(t\right)\right)=r^{o}\left(t\right)+\sum_{s(t)}p^{o}\left(t,t+1\right)\left(\max_{o(t)}Q_{\pi }^{*}\left(s\left(t+1\right),o\left(t+1\right)\right)\right),$$

In the next subsection, we use the above derivation to describe the training procedure.

### 4.5. Training Algorithm of the Proposed OHDRL Method

**Figure 3:**
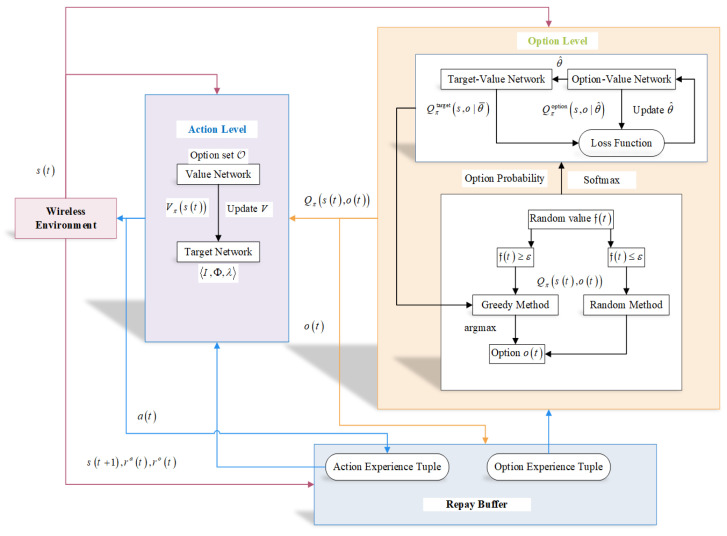
Structure of the proposed OHDRL.

In this subsection, we describe the training algorithm of the proposed OHDRL framework, which is shown in Figure 3. As can be observed, the proposed OHDRL includes two parts: one for options and the other for actions. Each part relies on two fully connected DNNs to stabilize the learning.

Target-value network $Q_{\pi }^{\mathrm{target}}\left(s,o|\bar{\theta }\right)$ is used to estimate the optimal option value function, with $\bar{\theta }$ indicating the target-value network’s parameters. Similar to this, the option-value network $Q_{\pi }^{\mathrm{option}}\left(s,o|\hat{\theta }\right)$ sets the value function of the current option-state, with $\hat{\theta }$ indicating the option-value network’s parameters. A replay buffer is used to store the transition experience $\left\{s(t),o(t),a(t),r(t),s(t+|o(t)|)\right\}$. Then, by using a mini-batch, samples from the replay buffer are used to train the DNNs. The loss function to evaluate the proposed model is given by

(50) $$L\left(\theta \right)=E\left[{\left(r\left(t\right)+\gamma \max_{o(t+1)}Q_{\pi }^{\mathrm{target}}\left(s\left(t+1\right),o\left(t+1\right);\bar{\theta }\right)-Q_{\pi }^{\mathrm{option}}\left(s\left(t\right),o\left(t\right);\hat{\theta }\right)\right)}^{2}\right],$$

where γ is a discount parameter. By using the gradient descent method, the network’s parameters are updated as follows

(51) $$\theta_{\mathrm{new}}=\theta_{\mathrm{old}}-\eta \nabla_{\theta }L\left(\theta \right),$$

where η is the learning rate, $\theta_{\mathrm{old}}$ and $\theta_{\mathrm{new}}$, respectively, indicate the network parameters before and after the update of the option-value network. The network parameters are continuously updated until the following condition is satisfied:

(52) $$Q_{\pi }^{\mathrm{option}}\left(\cdot |\hat{\theta }\right)\approx Q_{\pi }^{\mathrm{target}}\left(\cdot |\bar{\theta }\right)\approx Q_{\pi }^{*}.$$

The optimal option is obtained using a ε−greedy algorithm with random probability, 0⩽ε⩽1. Hence, the option is selected as

(53) $$o\left(t\right)=\underset{o\left(t\right)}{\arg\max}Q_{\pi }^{\mathrm{option}}\left(s\left(t\right),o\left(t\right);\hat{\theta }\right).$$

Before choosing the next option, the current option is executed for a certain period of time in different states, depending on the option type. The training procedure is repeated until the end of the task. Algorithm 1 presents the details of the procedure.
**Algorithm 1:** OHDRL Algorithm1:**Input:** Episodes E, training steps T, batch size, structure of option-state neural network,a.2:**Output:** Optimal option set, target-value network parameters.3:**Initialization:** Evaluation option-state network $Q_{\pi }^{\mathrm{option}}$ with $\hat{\theta }$, target option-state network $Q_{\pi }^{\mathrm{target}}\left(s,o|\bar{\theta }\right)$ with $\bar{\theta }$, options flag F, and replay buffer with capacity C.4:**for** e=1:E **do**5:    Get initial state $s_{0}$ and set $s\leftarrow s_{0}$.6:    **for** t=1:T **do**7:        **if** F=1 **then**8:           **if** c<C **then**9:                 Generate random value $f\left(t\right)$;10:               **if** $f\left(t\right)⩽\varepsilon$ **then**11:                   Selected option randomly;12:                   Update a via (47);13:               **else**14:                   Use option via greedy method;15:               **end if**16:               Set F=O;17:           **end if**18:        **else**19:           Execute current option;20:           Choose action $a\left(t\right)$ based on $\Phi \left(a\left(t\right)|s\left(t\right)\right)$;21:           Calculate the reward for the current option $r^{o}\left(t\right)$ and the state $s\left(t+1\right)$;22:           **if** $\lambda \left(s\left(t\right)\right)$ terminates **then**23:               Set F=1;24:               Calculate the reward for the current option $r^{o}\left(t\right)$ and the state $s\left(t+1\right)$;25:               Store the transitions $\left\{s\left(t\right),o\left(t\right),a\left(t\right),r\left(t\right),s\left(t+\left|o\left(t\right)\right|\right)\right\}$ to the replay buffer;26:           **end if**27:        **end if**28:        Get random samples from the replay buffer;29:        Calculate the loss function via (50);30:        Update the network’s parameters (51);31:    **end for**32:**end for**


### 4.6. Complexity Analysis

In this work, we formulate the joint UAV trajectory design, transmit power allocation, content fetching, and content placement problem as a constrained energy consumption minimization problem. To tackle the NP-hard problem, we formulate the problem as an SMDP and the proposed OHDRL framework.

Note that to conduct the proposed OHDRL, we need to initialize the DNNs and replay buffers and then perform two rounds of iterations in various episodes and steps. The complexity analysis of the proposed algorithm can be analyzed below. Let $T_{D}$ denote the time consumed for DNN initialization, let $T_{B}$ denote the time required to initialize the replay buffer in each episode, and let $T_{c}$ represent the time consumed for computation operations in each step. In addition, we denote $T_{E}$ and $T_{S}$ as the number of training episodes and steps, respectively. The total time complexity of the OHDRL algorithm can be expressed as $T_{\mathrm{OHDRL}}=T_{D}+T_{E}\cdot \left(T_{B}+T_{S}\cdot T_{c}\right)$.

Since the OHDRL algorithm employs a multi-agent framework, the complexity is linear with respect to the number of UAV agents M. Therefore, the final computational complexity is $O\left(T_{\mathrm{OHDRL}}\right)=O\left(M\cdot T_{E}T_{S}\right)$.

## 5. Simulation Results

In this section, the effectiveness of our proposed OHDRL-based algorithm is evaluated using values from the numerical simulations. The size of the simulation region was set to 0.35 km × 0.4 km, with a number of users randomly distributed within the area. The number of UAVs was set to M = 4, with all UAVs flying at a given altitude of H = 200 m. The number of contents was set to K=5, with the content sizes given by F={72,81,88,76,103}. At each time slot, each user randomly demands one content item from the content library. The DNNs were implemented based on the PyTorch framework, and the training processes were executed on a GPU, Gen Intel i5-12400. We utilized five fully connected hidden layers, with sizes of [400, 300, 256, 128, 64] for the prediction network and [800, 600, 512, 256, 128] for the target network. A list of additional parameters used in the simulation is provided in Table 3.

**Figure 4:**
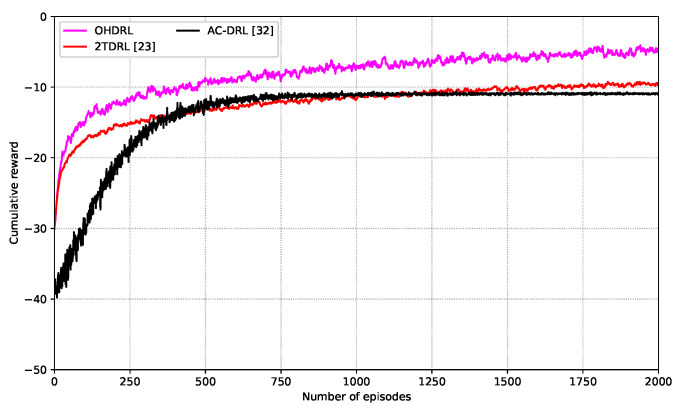
Cumulative reward comparison of the OHDRL, 2TDRL, and AC-DRL schemes.

Figure 4 illustrates the cumulative reward achieved by the proposed OHDRL framework compared with the 2TDRL and AC-DRL schemes over 2000 training episodes. From the figure, it can be observed that the OHDRL framework demonstrates superior performance, achieving faster convergence and higher cumulative reward compared to the baseline methods. This improvement can be attributed to the hierarchical structure embedded in OHDRL, which allows for a more effective balance between exploration and exploitation. From the figure, we can observe that the 2TDRL framework exhibits slower convergence and suboptimal results compared to OHDRL. The reason is that the separation of time scales in the 2TDRL framework may lead to slower convergence since changes at one scale can impact the other. In contrast, the AC-DRL approach exhibits the lowest cumulative reward and slower learning progress, indicating its limited ability to handle the complexities of joint trajectory design and content fetching. These results demonstrate the effectiveness of the proposed OHDRL algorithm. It can also be seen that during the training phase, the cumulative reward changes, but the algorithm eventually reaches convergence. Then, during the evaluation phase, the joint content fetching and UAV trajectory strategy can be obtained.

**Figure 5:**
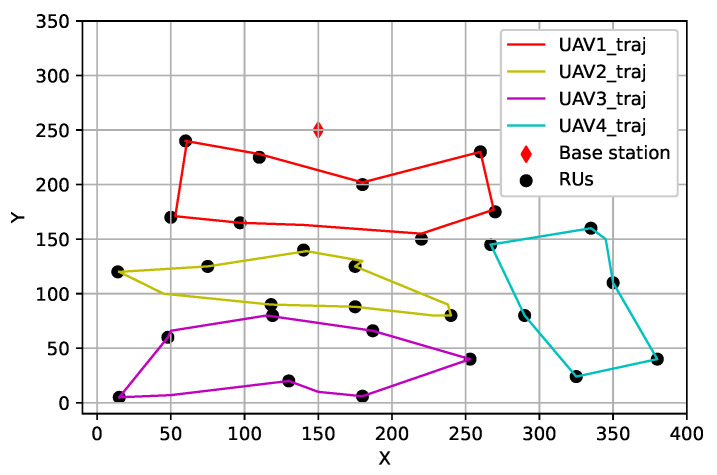
UAV trajectory of the proposed OHDRL-based content fetching.

The trajectories of the four UAVs produced by the proposed OHDRL-based algorithm are displayed in Figure 5. It is seen that the four UAVs fly in close proximity to the RUs they are serving and hover above each of them. Under the constraints of content fetching, we can observe from the figure that one UAV can serve multiple RUs, and one RU can be served only by one UAV.

**Figure 6:**
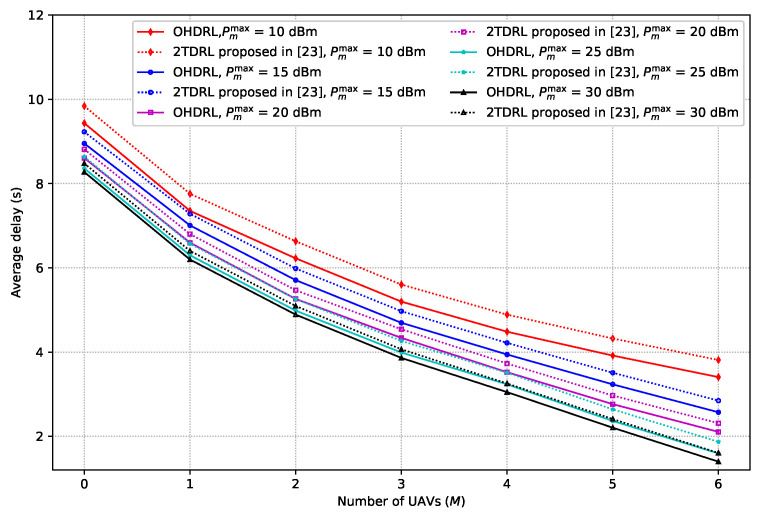
Average delay versus the number of UAVs for different maximum transmit powers of UAVs.

Figure 6 depicts the average delay versus the number of UAVs for various maximum transmit powers of UAVs. In the figure, we compare the performance of the algorithm proposed in this paper with the one proposed in [23]. We set the maximum transmit power of the UAVs as 10 dBm, 15 dBm, 20 dBm, 35 dBm, and 30 dBm. The figure shows that the average delay decreases as the number of UAVs increases. This is due to the possibility that more UAVs may cooperate to serve the RUs, which reduces the average delay. We can also observe that for different maximum transmit powers of the UAVs, the highest maximum transmit power results in better performance. The reason is that a higher maximum transmit power offers more flexibility in selecting the optimal power, thus resulting in a smaller delay. By contrasting the results obtained from our proposed algorithm with the one proposed in [23], we can observe that our proposed method technique provides less delay. The reason is that the decoupling of the usage of different levels during the learning process, i.e., option level and action level, decreases the impact of the large action space on the solution complexity, resulting in better performance.

**Figure 7:**
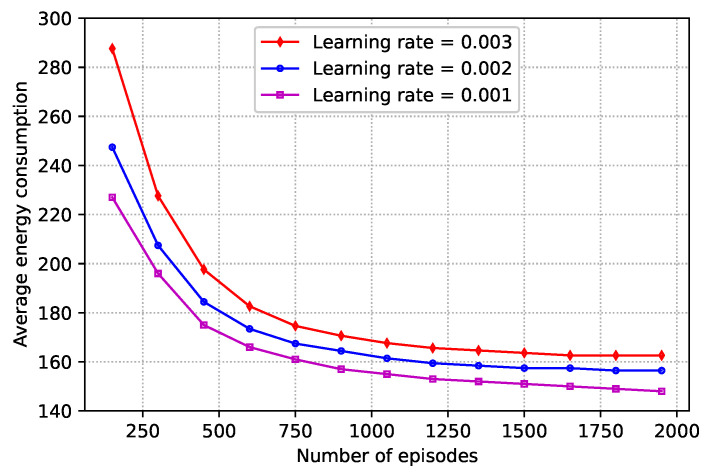
Average energy consumption versus the number of episodes.

In Figure 7, the average energy consumption versus the number of episodes for various learning rates is plotted. As can be observed from the figure, the average energy consumption decreases as the number of episodes increases. This is because various learning rates have different impacts on the performance of the proposed OHDRL; a lower learning rate can result in a more stable convergence, which lowers the requirement for needless computations and, as a result, lowers energy consumption. It can also seen that the trade-off between learning rate, convergence speed, and final accuracy in the OHDRL framework involves balancing stability and optimization performance.

**Figure 8:**
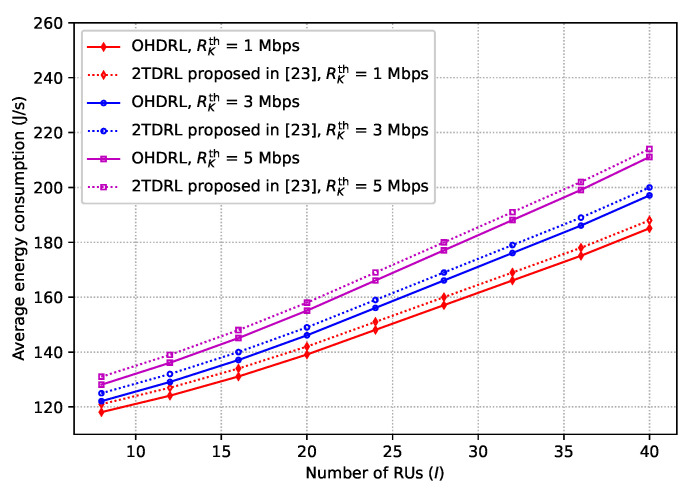
Average energy consumption versus the number of nodes.

Figure 8 illustrates the energy consumption performance of the proposed OHDRL-based algorithm under different numbers of RUs and rate requirements ($R_{k}^{\mathrm{th}}$). As shown in the figure, energy consumption increases as the number of RUs increases. This is because, with a larger number of RUs, the UAVs must cover longer trajectories to service all the RUs. This results in higher propulsion energy costs and greater energy consumption due to the extended flight path of the UAV, leading to a higher average energy consumption. Moreover, we observe that the energy consumption also increases as the rate requirement, $R_{k}^{\mathrm{th}}$, increases. The reason is that a higher rate requirement demands the transmission of larger data volumes in a shorter amount of time, necessitating stronger signal powers. The increased signal power leads to higher energy consumption because stronger transmitters are required, and maintaining reliable communication links becomes more energy-intensive, particularly in the presence of environmental factors or interference. In particular, the proposed OHDRL-based algorithm outperforms the method of [23] in terms of energy efficiency. This is primarily due to the multi-level learning approach embedded in OHDRL, which optimizes UAV trajectories and task allocations more efficiently, thus reducing the overall energy consumption for both low and high data rate requirements.

**Figure 9:**
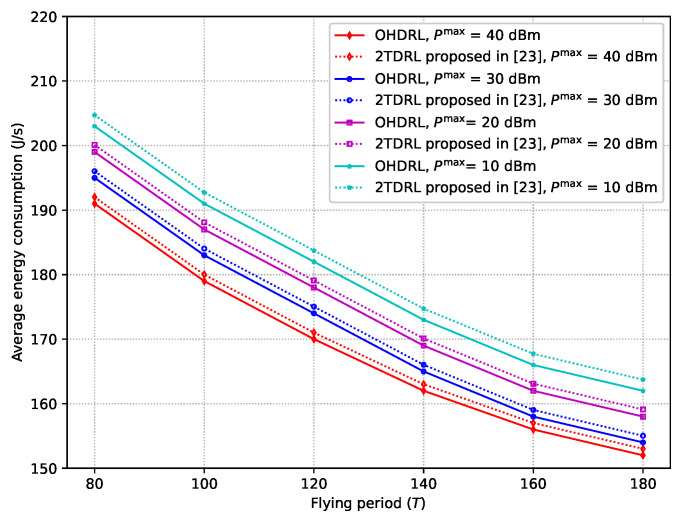
Average energy consumption versus flying period.

In Figure 9, the average energy consumption versus the flying period of UAVs for various transmit powers of a BS are plotted. In this figure, we compare the performance of the method proposed in this research with the one proposed in [23]. The graphic shows that while the flying time increases, the average energy consumption decreases. This reduction is due to the ability of UAVs to optimize their flight trajectories over a longer period. With more time, the UAVs can plan more energy-efficient routes, which reduces the need for abrupt adjustments or inefficient flight paths. The extended flying period allows for smoother, more gradual maneuvers, reducing the overall energy required for propulsion and improving task completion efficiency. In addition, the figure illustrates that an increase in the maximum BS transmit power results in a decrease in the overall energy consumption. This occurs because a higher transmit power ensures that data can be transmitted with greater efficiency, reducing the need for the UAVs to expend additional energy compensating for weak or fluctuating signals. With a stronger signal, the UAVs experience fewer communication failures and retransmissions, which, in turn, minimizes the energy required for flight path adjustments and maintains the link. By comparing the results obtained from the proposed OHDRL and 2TDRL in [23], we can see that the average performance gap between the two algorithms is about 5.91%.

**Figure 10:**
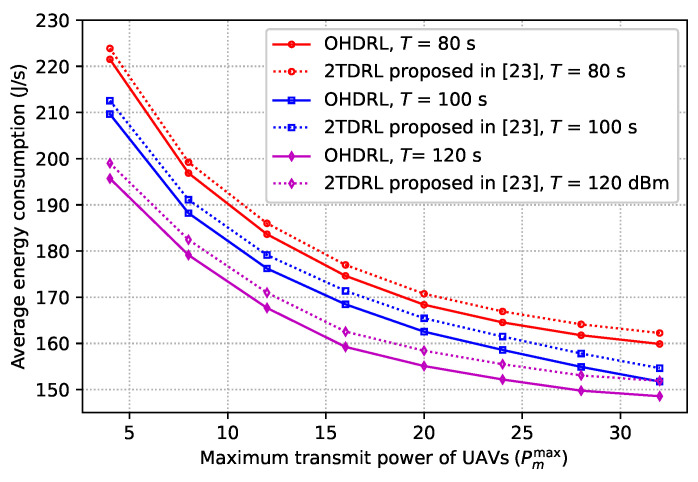
Average energy consumption versus maximum transmit power of UAVs.

Figure 10 depicts the average energy consumption versus the maximum transmit power of UAVs for various flying periods. The figure presents a comparison between the method described in this work and the one proposed in [23]. The graph indicates a decrease in average energy consumption with an increase in UAV maximum transmit power. This is due to the fact that boosting UAV maximum transmit power provides flexibility in determining the ideal transmit power, which lowers energy consumption. Furthermore, as the graphic illustrates, the average energy consumption decreases with increasing flying time. The reason is that a longer flight time allows the UAVs to adjust their trajectories appropriately. Therefore, by efficiently enhancing the channel conditions between the RUs and UAVs, energy consumption can be decreased. By comparing the performance obtained from our proposed method and the one proposed in [23], we can observe that our proposed algorithm offers low energy consumption.

**Figure 11:**
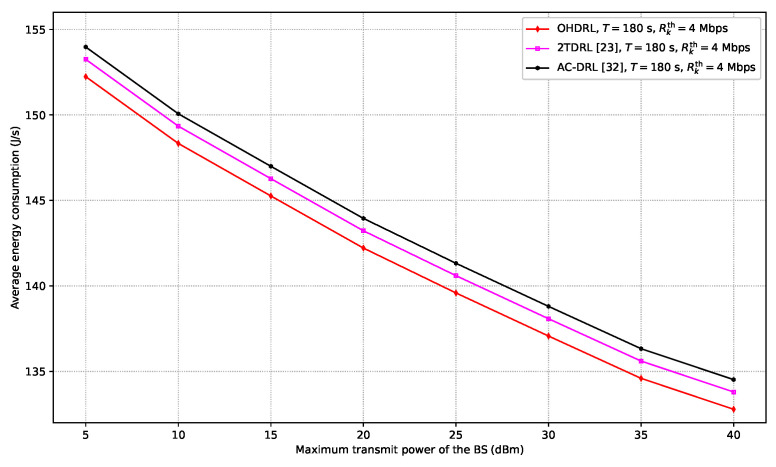
Average energy consumption versus maximum BS transmit power, $P^{max}$, and a fixed $P_{m}^{max}$.

In Figure 11, the performance of the proposed OHDRL framework is compared with the 2TDRL and AC-DRL schemes in terms of average energy consumption across varying maximum BS transmit power. The analysis is conducted under the constraints of a time period of T=180 s and a rate threshold of $R_{k}^{\mathrm{th}}=4$ Mbit/s. The results show that the average energy consumption of the OHDRL, 2TDRL, and AC-DRL frameworks decreases as the maximum transmit power of the BS increases, indicating their ability to optimize resource allocation under a higher maximum transmit power. This is because higher transmit power may enable the system to operate at lower transmission rates or fewer required transmission periods, thus contributing to a reduction in energy consumption. Additionally, as shown, OHDRL achieves the lowest average energy consumption across all $P^{\max}$ levels, indicating its superior efficiency in managing energy resources. 2TDRL follows closely, demonstrating a slightly higher energy consumption compared to OHDRL, while AC-DRL consistently exhibits the highest energy consumption, suggesting less energy-efficient performance. The OHDRL framework consistently outperforms both 2TDRL and AC-DRL; this is because OHDRL leverages a hierarchical decision-making structure that enables a more intelligent allocation of resources.

## 6. Conclusions and Future Work

### 6.1. Conclusions

In this paper, we have examined the joint UAV trajectory planning, content fetching, power allocation, and content placement problem in a UAV-enabled network. The system’s overall energy consumption was computed, and the joint UAV trajectory design, content fetching, power allocation, and content placement problem was formulated as an energy consumption minimization problem. To address the complexity of the problem, we proposed an OHDRL algorithm, which effectively leverages a hierarchical policy to tackle high-dimensional state and action spaces. Through extensive simulations, we evaluated the performance of the proposed algorithm under various parameters, such as the number of RUs, the number of UAVs, the UAV flying period, the maximum power of the UAVs, and the rate requirement. The results indicate that the proposed OHDRL-based technique achieves a significant reduction in energy consumption compared to existing methodologies, demonstrating its effectiveness in optimizing resource allocation and improving network performance. Finally, The broader implications of this work lie in its potential to enhance energy efficiency and scalability in UAV-enabled networks, which is crucial for supporting the growing demands of modern wireless networks. By reducing energy consumption, the proposed approach contributes to sustainable network operation and supports applications in IoT, disaster recovery, remote area connectivity, etc.

### 6.2. Future Work

In this work, we address the challenges of joint UAV trajectory planning, content fetching, power allocation, and content placement under the assumption of static RUs and known environmental parameters. While our framework achieves significant energy efficiency improvements, there are several important directions for future research:

This work considers static RUs, but in practical applications, such as UAV-assisted vehicular networks, the targets or RUs may be mobile. For example, the movement of vehicles could complicate trajectory planning and content delivery. Future work could investigate the proposed framework in scenarios with mobile RUs, incorporating real-time trajectory adjustment and dynamic scheduling strategies to handle mobility challenges.

Furthermore, in our current system model, environmental parameters, such as channel conditions and RU demands, are assumed to be static and known in advance. However, real-world scenarios often involve dynamic and uncertain conditions. For instance, sudden changes in RU demands or interference levels may require real-time adaptability. Future research could focus on designing robust and adaptive algorithms capable of handling such dynamic environments and uncertainties.

In addition, to further enhance the energy efficiency of UAV networks, incorporating renewable energy sources or energy harvesting mechanisms is a logical next step. For instance, UAVs equipped with solar panels or energy-recycling capabilities could sustain longer operations. The joint optimization of energy harvesting, storage, and resource allocation would present an interesting challenge for future studies.

## Figures and Tables

**Table 1:** Table of the main notations.

Parameter	Symbol
I	Number of RUs
M	Number of UAVs
N	Total number of time slots
$q_{m}\left(t\right)$	The locations of U*_m_* at time slot t
$q_{i}$	The co-ordinates of RU*_i_*
$q_{b}$	The co-ordinates of BS
$h_{m}\left(t\right)$	BS-UAV channel gain at time slot t
$h_{m,i}\left(t\right)$	UAV-RU channel gain at time slot t
$d_{m}\left(t\right)$	Distance between BS and U*_m_*
$d_{m,i}\left(t\right)$	Distance between U*_m_* and RU*_i_*
$R_{m}\left(t\right)$	Data transmission rate between BS and U*_m_*
$R_{m,i}\left(t\right)$	Data transmission rate between U*_m_* and RU*_i_*
$P_{m}^{t}\left(t\right)$	Transmit power of BS
$P_{m,i}^{t}\left(t\right)$	Transmit power of U*_m_*
$P_{m}^{f}\left(t\right)$	Power consumption of U*_m_*
$v_{m}^{\max}$	Maximum speed of U*_m_*
*K*	Number of contents
$\delta_{m,k}$	Content placement variable of U*_m_* for content k
$\alpha_{m,i}\left(t\right)$	Content fetching variable of RU*_i_* from U*_m_*
$\phi_{m,k}\left(t\right)$	Content fetching variable of U*_m_* for content k
$E^{t}\left(t\right)$	Transmission energy consumption
$E^{f}\left(t\right)$	Flight energy consumption of UAVs
$E^{h}\left(t\right)$	Hovering energy consumption
$E_{m}^{t}\left(t\right)$	Transmission energy consumption of U*_m_*
$E_{m,i}^{t}\left(t\right)$	Transmission energy consumption of U*_m_* while delivering contents to RU*_i_*
$E_{b,m}^{t}\left(t\right)$	Transmission energy consumption of BS while delivering contents to U*_m_*
$S_{i}\left(t\right)$	Remaining data that RU*_i_* fetches from UAVs at time slot t
${\bar{S}}_{m}\left(t\right)$	Remaining data that U*_m_* fetches from BS at time slot t
$T_{m,i}\left(t\right)$	Transmission delay from U*_m_* to RU*_i_* at time slot *t*
$T_{m}\left(t\right)$	Transmission delay from BS to U*_m_* at time slot *t*

**Table 2:** Summary of the dimensions of the state, action, and option variables.

Variable	Description	Dimension
States		
$S_{1}\left(t\right)$	Co-ordinates of the UAVs	2M
$S_{2}\left(t\right)$	Channel state information	M(I+1)
$S_{3}\left(t\right)$	Remaining amount of data of UAVs and RUs	I+M
$S_{4}\left(t\right)$	Caching capacity state	M
Actions		
$a_{1}\left(t\right)$	Flying distance and direction	2M
$a_{2}\left(t\right)$	Flying and hovering variable	M
$a_{3}\left(t\right)$	Transmit power of the BS and $U_{m}$	M(I+1)
Options		
$\alpha_{m,i}\left(t\right)$	Content fetching variable of RU*_i_*	M×I
$\phi_{m,k}\left(t\right)$	Content fetching variable of U*_m_*	M×K
$\delta_{m,k}$	Content placement variable of U*_m_*	M×K

**Table 3:** Simulation parameters.

Parameter	Value
Carrier frequency ($f_{c}$)	28 GHz
Link Bandwidth (B)	5 MHz
Maximum transmit power of the BS ($P^{\max}$)	40 dBm
Maximum transmit power of U*_m_* ($P_{m}^{\max}$)	30 dBm
Noise power ($\sigma^{2}$)	−114 dBm
Path loss coefficient (α)	2.7
Maximum speed of U*_m_* ($v_{m}^{\max}$)	20 m/s
Number of UAVs	4
Number of contents (K)	5
Set of the size contents (F*_k_*)	{72,81,88,76,103}
Maximum cache capacity	{150,80,90,110}
Blade profile power	0.12
Induced power	0.18
Default number of RUs	24
Learning rate (α)	0.001
Discount parameter (γ)	0.99
Time slots (τ)	20 s
Replay buffer size	100,000
Number of episodes	2000
Steps per episode	2000
Batch size	64
Optimizer	Adam

## Data Availability

The original contributions presented in this study are included in the article. Further inquiries can be directed to the corresponding authors.
